# The negative compatibility effect: A case for self-inhibition

**DOI:** 10.2478/v10053-008-0027-y

**Published:** 2008-07-15

**Authors:** Friederike Schlaghecken, Laura Rowley, Sukhdev Sembi, Rachel Simmons, Daniel Whitcomb

**Affiliations:** Department of Psychology, University of Warwick, Coventry, UK

**Keywords:** masked priming, negative compatibility effect, inhibition

## Abstract

In masked priming, a briefly presented prime stimulus is followed by a mask, which in turn is followed by the task-relevant target. Under certain conditions, negative compatibility effects (NCNCEs) occur, with impaired performance on compatible trials (where prime and target indicate the same response) relative to incompatible trials (where they indicate opposite responses). However, the exact boundary conditions of NCEs, and hence the functional significance of this effect, are still under discussion. In particular, it has been argued that the NCE might be a stimulus-specific phenomenon of little general interest. This paper presents new findings indicating that the NCE can be obtained under a wider variety of conditions, suggesting that it reflects more general processes in motor control. In addition, evidence is provided suggesting that prime identification levels in forced choice tasks – usually employed to estimate prime visibility in masked prime tasks – are affected by prior experience with the prime (Exp. 1) as well as by direct motor priming (Exp. 2 & 3).

## Introduction

For any complex organism, the successful control of behaviour requires the ability to quickly detect potentially relevant changes in the environment, and to adjust ongoing motor processes in response to these changes. These fast and usually small-scale modifications are generally assumed to be ‘automatic’ (i.e., independent of online intentional or top-down control), because they will be elicited a) even by response-irrelevant stimuli or stimulus features, and b) even when they are detrimental to the current behavioural goals. For example, in the Erikson flanker task, behavioural and electrophysiological evidence indicates that once the required stimulus-response mappings have been established, motor activation is not only triggered by the response-relevant central target stimulus, but also by the response-irrelevant flanking distractor stimuli (e.g., Smid, Mulder, & Mulder, 1990). If these flankers indicate a different response than the target (e.g., if the target requires a left-hand response, but the flanking stimuli are associated with a right-hand response), then a *response conflict* results, which will increase reaction times (RTs) and error rates.

This might suggest that such automatic response activations are generally disadvantageous and should be suppressed at all costs – however, we need to keep in mind that the flanker task and similar experimental paradigms represent highly artificial situations in that the response-relevant and -irrelevant stimuli are always defined in advance (e.g., via their spatial location). In a natural environment, in contrast, *every* stimulus is potentially response relevant and thus should be given access to the motor system. Obviously, though, even in a natural environment not all stimuli do, in fact, require an overt response. Consequently, control mechanisms need to be in place to ensure that behavioural modifications remain in line with the overall behavioural goals. Such control mechanisms have traditionally been regarded as ‘voluntary,’ ‘top-down,’ or ‘goal-driven’ (as opposed to the initial ‘involuntary,’ ‘bottom-up,’ or ‘stimulus-driven’ motor activation), or in other words, as ‘controlled’ as opposed to ‘automatic’ (e.g., Band & van Boxtel, 1999). However, these distinctions are far less clear-cut than one might expect – after all, provided that we do not wish to invoke the notion of a ‘homunculus’ in the brain, even the most high-level control functions have to be instantiated by automatic neural processes (for a recent discussion of these issues, see, e.g., Hommel, 2007).

The masked prime paradigm has been used to directly investigate such ‘automatic control.’ In a typical task, a briefly presented prime stimulus is followed by a mask, which in turn is followed by the task-relevant target stimulus. On any given trial, the prime might be a stimulus associated with the same response as the subsequent target (compatible trial), a stimulus associated with the opposite response (incompatible trial), or a stimulus not associated with any response at all (neutral trial). Although the prime might remain below the observer’s threshold of conscious perception due to its brief presentation and subsequent masking, it can nevertheless trigger an activation of its corresponding motor response, as evidenced by electrophysiological and haemodynamic measures (e.g., Dehaene et al., 1998; Eimer, 1999; Eimer & Schlaghecken, 1998; Leuthold & Kopp, 1998). If motor activation in response to the target occurs while the prime-induced activity is still present, then this will result in *positive compatibility effects* (PCEs) with behavioural benefits on compatible and costs on incompatible trials relative to neutral trials (e.g., Aron et al., 2003; Dehaene et al., 1998; Eimer, 1999; Schlaghecken & Eimer, 1997, 2000; Seiss & Praamstra, 2004). However, if motor activation in response to the target occurs later, then the reverse pattern is observed, that is, *negative compatibility effects* (NCEs) with behavioural benefits on incompatible trials and costs on compatible trials relative to neutral trials occur (e.g., Aron et al., 2003; Eimer, 1999; Klapp & Haas, 2005; Klapp & Hinkley, 2002; Lingnau & Vorberg, 2005; Schlaghecken & Eimer, 1997, 2000, 2002, 2006).

We have argued that this latter effect reflects a low-level and automatic process of inhibitory motor control (Schlaghecken, Bowman, & Eimer, 2006; Schlaghecken & Eimer, 2002, 2006), which acts as an ‘emergency brake’ mechanism to stop early motor activations that are no longer supported by sensory evidence from affecting overt motor output. According to this view, early stages of the motor system employ an opponent processing design, whereby stimulus-induced activity in a response channel’s ‘on node’ results in correspondingly increased activity in its inhibitory ‘off node.’ As long as on-node activity remains supported by sensory input, off-node activity will be counterbalanced and thus will be of no consequence. If, however, on-node activity suddenly loses its supporting sensory input, then off-node activity might ‘take over’ and rapidly inhibit the initially activated response (reflected in behavioural costs on compatible trials). In a two-alternative choice RT task, this inhibition of one response alternative will cause disinhibition of the opposite alternative, resulting in behavioural benefits on incompatible trials (Schlaghecken et al., 2006).

This concept of low-level motor self-inhibition is theoretically interesting insofar as it represents an example of a complex inhibitory control process that is entirely automatic (i.e., not top-down driven by central executive mechanisms in the prefrontal lobes, which are traditionally assumed to mediate inhibitory control; see Band & van Boxtel, 1999; Faw, 2003). However, the exact conditions required to obtain NCEs, and hence the functional significance of this effect, are still under discussion. For instance, Lleras and Enns (2004) and Verleger, Jaśkowski, Aydemir, van der Lubbe, and Groen (2004) have argued that the NCE does not reflect low-level and automatic self-inhibition, but rather a mask-induced activation of the opposite response. Empirically, this hypothesis is based on the observation that in a typical masked prime study, primes and targets are directional arrow stimuli presented at fixation, and masks are constructed from potentially task-relevant elements (e.g., diagonal lines). Lleras and Enns and Verleger et al. argue that these conditions (or a critical combination of some of them) are necessary to obtain NCEs, and that therefore the NCE might be a stimulus-specific phenomenon of little general importance: If the mask is in some way similar to the stimulus indicating the opposite response, and therefore triggers this response (i.e., if the NCE simply reflects automatic response activation followed by another automatic response activation), then there is nothing conceptually new or interesting in this effect. If, on the other hand, NCEs can be obtained even when these criteria are not met, then this would support the self-inhibition hypothesis. The present paper presents three experiments that indicate that the NCE does not depend on arrow stimuli and potentially task-relevant masks, suggesting that it reflects a more general and possibly conceptually interesting phenomenon in motor control.

## Experiment 1: Non-Arrow Primes and Targets

This experiment aimed to confirm that NCEs can be obtained with non-arrow stimuli. In most previous experiments, primes and targets have been arrows or arrow-like stimuli (e.g., ‘<<’ or ‘<>’), and it has been argued that arrows are ‘special’ in MP situations (Jaśkowski & Ślósarek, 2007; Verleger et al., 2004; Verleger, Görgen, & Jaśkowski, 2005). Although NCEs have also been found with non-arrow primes and targets, most of these stimuli consisted of intersecting straight lines of different relative position or orientation (e.g., Eimer, 1999; Verleger et al., 2005). Moreover, the corresponding masking stimuli also consisted of intersecting straight lines, thus potentially containing task-relevant features. If these features would trigger a response opposite to the primed response, then this process – rather than low-level motor inhibition – could give rise to NCEs (Lleras & Enns, 2004; Verleger et al., 2004). Although at present the evidence suggests that NCEs do in fact reflect self-inhibition of motor responses triggered by successfully masked primes (Klapp, 2005; Schlaghecken & Eimer, 2006; Sumner, in press), the question remains whether NCEs can be obtained with a different type of stimulus.

The present experiment employed circles with a small gap on either the left or right side as prime and target stimuli (inspired by Di Lollo, Enns, & Rensink, 2000), to which participants had to make a corresponding left- or right-hand response. The mask consisted of an array of interlinked complete circles. These stimuli differ from arrow primes and targets and from their corresponding pattern masks in three important respects. First, they differ at the feature level, as they are not composed of intersecting straight lines.1 Second, unlike directional arrows, they are not over-learned indicators of left and right responses. Finally, the mask does not contain the response-relevant feature (i.e., the ‘gap’). Finding NCEs with these stimuli would thus strengthen the argument that NCEs reflect a general phenomenon in motor control. In order to obtain an estimate of the masked primes’ visibility, and thus to facilitate comparability of the present and previous studies, the experiment also included a forced choice prime identification task.

### Method

#### Participants

Thirty volunteers (11 male), aged 18–38 years (M = 23.1), participated in the experiment. All but two participants were right-handed, and all had normal or corrected-to-normal vision. One participant was excluded from further analysis because of excessive error rates (more than 15% errors in the masked prime task), and two further participants were excluded because they were able to consciously perceive even 17-ms primes (more than 70% correct responses in the forced choice prime identification task). This left 9 participants in group A (17 ms prime duration), 8 participants in group B (33 ms prime duration), and 10 participants in group C (50 ms prime duration). Eight additional volunteers (three male), aged 19–27 years (M = 21.4), participated in a control condition (group D).

#### Stimuli and apparatus

**Figure 1.**
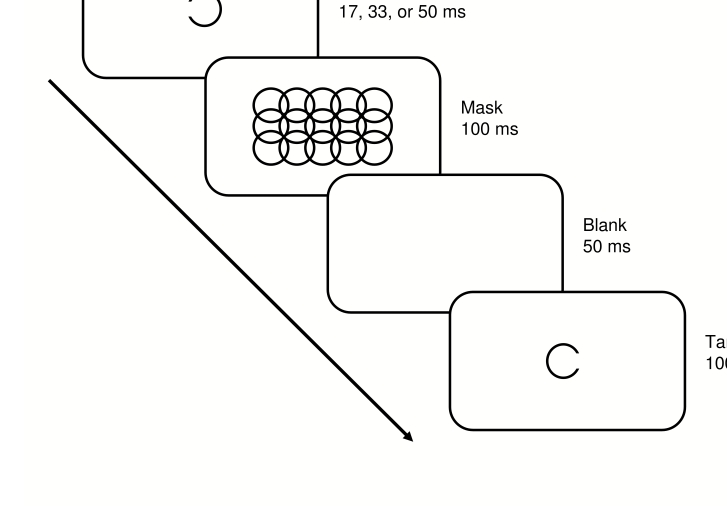
Stimulus- and trial-structure in Experiment 1.

Primes and targets were single circles with a diameter of 0.75° of visual angle. Target circles had a gap on either the left or the right side (see Figure 1), indicating a left- or right-hand response, respectively. Prime circles either had a left or right gap or were complete. Masks were 15 complete circles of the same dimensions, arranged in three rows of five, each circle overlapping with its neighbours, resulting in a rectangular array of approximately 1.5° x 3° of visual angle. All stimuli were presented in black on a white background at the centre of the screen.

#### Procedure

Participants were seated in a dimly lit room, facing a computer screen at a viewing distance of 100 cm. Their left and right index fingers rested on response keys attached to the armrests of the chair. The experiment consisted of a masked prime (MP) reaction time part and a forced choice (FC) prime identification task.

The MP part began with a 24-trial practice block, followed by 12 blocks of 60 trials each. Each trial began with the presentation of a prime circle, followed immediately by the mask for 100 ms. After mask offset, the screen remained blank for 50 ms, then a target was presented for 100 ms (see Figure 11). Prime duration was 17, 33, or 50 ms in groups A, B, or C, respectively, and 50 ms in group D. Primes were either compatible with the target (same-side gap), incompatible (opposite-side gap), or neutral (complete circles). Inter-trial-interval (ITI) was 1,650 ms. Participants were instructed to make a speeded left- or right-hand response to a gap on the left or right side of the target circle, respectively. Left and right targets, and compatible, incompatible, and neutral trials were randomized and appeared with equal probability in each block.

The FC part began with a 14-trial practice block, followed by one 60-trial block. Primes were presented for 17, 33, 50, 67, 83, or 100 ms and immediately followed by a 100-ms mask. No target stimuli were presented.2 Participants were instructed to respond to the primes in the same way they had responded to the targets in the MP part of the experiment (i.e., left- and right-hand responses to a gap on the left and right, respectively – consequently, no neutral primes were employed). They were informed that response speed was unimportant in this block, and that they should simply guess if they felt that they could not make an informed choice.

#### Data analysis

Repeated measures analyses of variance (ANOVAs) were computed on correct RTs and error rates for the between-subject factor Group (A – 17 ms, B – 33 ms, and C – 50 ms prime duration) and the within-subject factor Compatibility (compatible, neutral, incompatible) in the MP task. ANOVAs were computed on the percentage of correct responses in the FC task for between-subject factor Group and the within-subject factor Presentation Duration (17, 33, 50, 67, 83, and 100 ms). Greenhouse-Geisser adjustments to the degrees of freedom were performed where appropriate, and corrected p-values are reported throughout.

### Results

**Figure 2.**
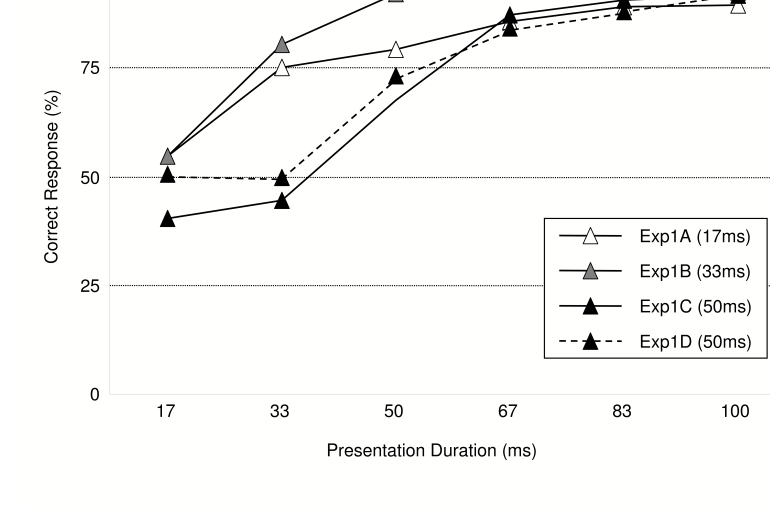
Forced Choice Identification Performance (percent correct) in Experiment 1.

Prime identification performance (Figure 2) increased with increasing prime duration, *F*(5, 120) = 89.14, *MSE* = 86.63, *p* < .001, ε = .619, and was generally lower for group C (50 ms) than for the other two groups, *F*(2, 24) = 5.22, *MSE* = 602.74, *p* = .013. Furthermore, these two effects interacted, *F*(5, 120) = 6.03, *MSE* = 86.63, *p* < .001, ε = .619, as the group differences were pronounced for short prime presentations, but virtually absent for durations of 67 ms or more. Follow-up analyses, conducted with one-sample t-tests, indicated that for groups A and B, prime identification was not significantly different from chance (50%) for 17-ms primes (54.8 and 54.6% correct, respectively, both *ts* < 1.6, both *ps* > .15), but was well above chance level for all other prime durations, all *ts* > 4.8, all *ps* < .001. Group C, in contrast, produced marginally significant below-chance identification with 17-ms primes, *t*(9) = 2.24, *p* = .052, near-chance identification with 33-ms primes, *t*(9) < 1.9, *p* > .10, and above-chance identification only with primes presented for 50 ms or more, all *ts* > 3.5, all *ps* < .007.

Because of this unexpected finding, eight additional participants (group D) were tested under identical conditions as group C. The finding of chance-level perfor-mance with 33-ms primes was replicated. Identification performance in group D was at chance level for both 17- and 33-ms primes, both *ts* < 0.5, both *ps* > .8, and was significantly above chance for all other presentation durations, all *ts* > 3.2, all *ps* < .02.

**Figure 3.**
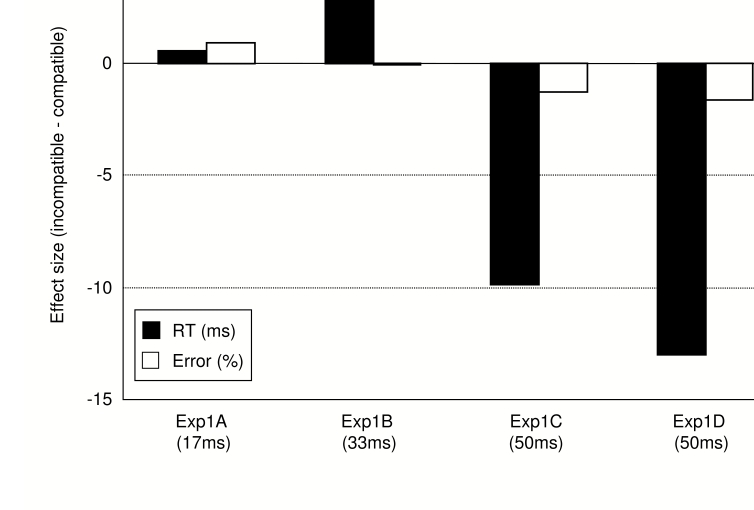
Priming effects (incompatible minus compatible) on reaction times (black) and error rates (white) in Experiment 1.

Behavioural results from the MP task are presented in Table 1. Reaction times showed neither a main effect of Compatibility nor a main effect of Group, both *F*s < 2.3, both *ps* > .11, but a highly significant interaction between these factors, *F*(4, 48) = 5.13, **MSE** = 25.67, *p* = .002. Follow-up ANOVAs, conducted for each group separately, confirmed that group C produced highly significant NCEs, *F*(2, 18) = 16.40, **MSE** = 16.52, *p* < .001, whereas no significant priming effects were obtained for group A and B, both *Fs* < 1.8, both *ps* > .22 (see Figure 3).

**Table 1.** Reaction times (ms) and Error Rates (%) on compatible, neutral, and incompatible trials, separately for each of the four groups in Experiment 1.

	RT (ms)			Error Rates (%)		
	Compatible	Neutral	Incompatible	Compatible	Neutral	Incompatible
Group A (17ms)	346	347	347	5.1	5	6
Group B (33ms)	357	356	361	5.3	4.5	5.2
Group C (50ms)	370	363	360	3.2	2.3	2
Group D (50ms)	353	342	340	2.8	1.6	1.1

Error rates in the MP task showed the same basic pattern as RTs. Although there was no significant main effect or interaction with error rates, all *Fs* < 2.3, all *ps* > .12, follow-up ANOVAs for the separate groups again revealed a significant NCE for group C, *F*(1, 18) = 4.64, *MSE* = 0.91, *p* = .024, and no priming effects for the other two groups, both *Fs* < 1. As expected, the additional 50-ms group D showed the same pattern of priming effects as group C, with NCEs that were significant for RTs, *F*(2, 14) = 12.38, *MSE* = 31.69, *p* = .003, one-tailed, and approached significance for error rates, *F*(2, 14) = 2.29, *MSE* = 2.59, *p* = .085, one-tailed.

### Discussion

The present results demonstrate that NCEs can be obtained with non-arrow primes and targets, and with masks that do not contain the response-relevant feature. However, the presentation conditions necessary to elicit NCEs with these stimuli differed from those of standard (arrow-based) MP experiments: NCEs occurred only when prime duration was 50 ms, which produced clearly above-chance identification performance, whereas with shorter durations (as employed in standard MP tasks), no reliable priming effects were obtained. In other words, a markedly higher perceptual strength was required for circle-primes compared with arrow-primes to elicit NCEs.3 Within the inhibition-threshold model developed by Schlaghecken and Eimer (2002), this might be taken as evidence that the low-level motor activation triggered by a gap in a circle is weaker than the activation triggered by an arrow: In this case, more sensory input would be needed to build up activation levels strong enough to require self-inhibition when the supporting sensory evidence is suddenly removed. However, although it might be plausible to assume that an over-learned arrow prime triggers strong activations more quickly than the present stimuli, this hypothesis must remain purely speculative, as there is as yet no way to independently assess the strength of low-level motor activation. Furthermore, it might be possible that instead of indicating conscious prime perception, the identification levels with 50-ms stimuli were due to direct motor priming effects (i.e., to the activation of the correct motor response by the masked prime). This issue will be discussed in more detail below (Experiments 2 and 3).

Another unexpected finding of Experiment 1 is the difference in FC prime identification performance between the experimental groups. Participants in groups A and B (with 17- and 33-ms primes, respectively, in the MP task) consistently identified 33-ms primes with above-chance accuracy, whereas participants in groups C and D (50-ms primes) equally consistently performed at chance level with this prime duration.4 This suggests that prior experience with the stimulus material in the MP task influences subsequent prime identification performance in the FC task. Although such a transfer effect has not been described previously in the MP literature, it is consistent with results from a recent perceptual learning experiment (Schlaghecken, Blagrove, & Maylor, in press). In this experiment, participants’ identification performance of 17-ms primes improved significantly even within one session when the masking stimulus was held constant across trials. It thus seems conceivable that in the present experiment, perceptual learning of ‘difficult’ short-duration primes occurred during the MP task for groups A and B, accounting for their above-chance identification of 33-ms primes (though not of 17-ms primes) in the subsequent FC task.

Participants in the two 50-ms groups, in contrast, did not encounter 33-ms (or shorter) primes in the MP task, and thus had no opportunity for perceptual learning of these primes. Consequently, they only identified longer-duration primes with more than chance accuracy in the FC task. Again, this reasoning has to remain speculative at present. Future studies will be needed to investigate such systematic carry-over effects in detail.

## Experiment 2: Irrelevant Masks and Non-Central Targets

 As noted in the Introduction, it has been argued that the NCE does not result from low-level motor inhibition, but merely reflects activation of the motor response opposite to the prime. More specifically, Verleger et al. (2004) argue that direct *perceptual interactions* between prime and mask trigger the opposite response, whereas Lleras and Enns (2004) suggest that in a continuous process of perceptual *object updating*, the most recent updates before target onset will be those of the mask’s potentially response-relevant features that are opposite to the prime, thus preparing the system to respond best to a stimulus containing these features. Importantly, a crucial requirement of both these models is that the mask contains potentially response-relevant features. Recently, Klapp (2005) and Schlaghecken and Eimer (2006) provided evidence that reliable NCEs can be obtained even when this is not the case. However, in their reply to Schlaghecken and Eimer (2006), Lleras and Enns (2006) argue that these findings still fail to support the notion of low-level motor self-inhibition, because primes, masks and targets were all presented at fixation. Instead, they propose that in addition to object updating (which triggers prime-opposite response activations with relevant masks), a process of ‘onset-triggered suppression’ (similar to Jaśkowski & Przekoracka-Krawczyk’s, 2005, ‘mask-triggered inhibition’) selectively inhibits the prime-induced response.5 According to the authors, there are three key components to the ‘object-updating-with-onset-triggered-suppression’ model: a) *geometrical similarity* of prime/target and mask (a mask that contains potentially response-relevant features in form of the same basic shapes as primes and targets triggers an activation of the opposite response via object updating), b) *temporal similarity* of prime/target and mask (a mask that is flashed only briefly, like a prime or a target, triggers selective inhibition of the prime-related response via onset-triggered suppression), and finally c) *spatial similarity* of prime/target and mask (both object updating and onset-triggered suppression affect priming most strongly when prime, mask and target appear at the same spatial location). Therefore, as long as all stimuli are presented at fixation, NCEs might be obtained even with irrelevant masks (i.e., via onset-triggered suppression only), although this effect will be smaller than when relevant masks are used (in which case effects of onset-triggered suppression and object updating are combined). However, when targets appear at a different position, no NCEs will be obtained with irrelevant masks, because temporal similarity alone – without geometrical and/or spatial similarity – is insufficient to elicit (sufficiently strong) onset-triggered suppression. 

Lleras and Enns (2006) present data that exactly follow the predicted pattern: With briefly flashed relevant masks, large NCEs occur when all stimuli appear at fixation, and smaller NCEs occur when targets are presented at a different position (above or below fixation). With briefly flashed *irrelevant masks*, small NCEs occur when all stimuli appear at fixation, but PCEs are obtained when targets are presented at a different position (see also Lleras & Enns, 2005). This last finding clearly conflicts with predictions derived from the self-inhibition account of NCEs. According to this model, target position should not make a difference to priming effects (other than trivial differences due to overall changes in reaction times) – all that should matter is that an initial strong motor response triggered by the prime is no longer supported by sensory evidence. 

However, we believe that there is an additional factor that might account for Lleras and Enns’ (2006) findings without violating the assumptions of the self-inhibition model. It should be noted that effective masking is far easier to accomplish when the mask contains elements similar to the to-be-masked stimulus (relevant mask) than when this is not the case (irrelevant mask). Thus it seems possible that in Lleras and Enns (2005, 2006), similar to the experiments in Lleras and Enns (2004), the irrelevant mask did not effectively remove the sensory evidence for the primed response from the motor system. If this was the case, then target position might make an important difference simply because it affects the continued availability of the prime-representation to the motor system: When targets are presented centrally (that is, ‘on top’ of the mask), they might act as an additional mask, cutting short the sensory evidence which supports the primed response and resulting in NCEs. In contrast, when targets are presented non-centrally (away from the mask), residual sensory evidence for the primed response might still be present in the system by the time the target-related response is activated, resulting in PCEs. 

In order to investigate this issue, we employed a ‘flicker mask’ procedure, which has been found to result in relatively (though not perfectly) effective masking with irrelevant masks (Schlaghecken & Eimer, 2006). Masked primes were presented at fixation, and targets were presented either centrally or non-centrally. According to the self-inhibition account of masked priming, NCEs of comparable size should be found with central and non-central targets. According to the object-updating-plus-onset-triggered-suppression account, in contrast, NCEs should be restricted to central targets, while non-central targets should result in PCEs, replicating Lleras and Enns’ (2006) results. The central-target condition of the present experiment is similar to the non-diagonal mask condition in Schlaghecken and Eimer (2006), except that prime duration was increased from 17 ms to 33 ms in order to investigate whether this would affect the size of NCEs. The critical question was whether priming effects would be similar for central and non-central targets.

### Method

#### Participants

Twenty volunteers (6 male), aged 19–30 years (M = 22.8), participated in the experiment. All but two participants were right-handed, and all had normal or corrected-to-normal vision. Four participants were excluded from further analysis because of excessive error rates (more than 10% errors in the masked prime task), and two further participants were excluded because they correctly identified more than 90% of the 33-ms primes in the FC task.

#### Stimuli and apparatus

Primes and targets were left- and right-pointing double arrows (‘<<’, ‘>>’), subtending a visual angle of approximately 1.0° x 0.5°. Masks were constructed from a 13 x 7 matrix, randomly filled with overlapping horizontal and vertical lines of different length, resulting in a rectangular array of about 2.5° x 1.5°. New random masks were generated on each trial, to avoid perceptual learning of the mask (Schubö, Schlaghecken, & Meinecke, 2001).

#### Procedure

Participants were seated in a dimly lit room, facing a computer screen at a viewing distance of approximately 100 cm. They were instructed to maintain central eye fixation. Response keys were the left and right SHIFT-keys of a standard qwerty computer keyboard.

**Figure 4.**
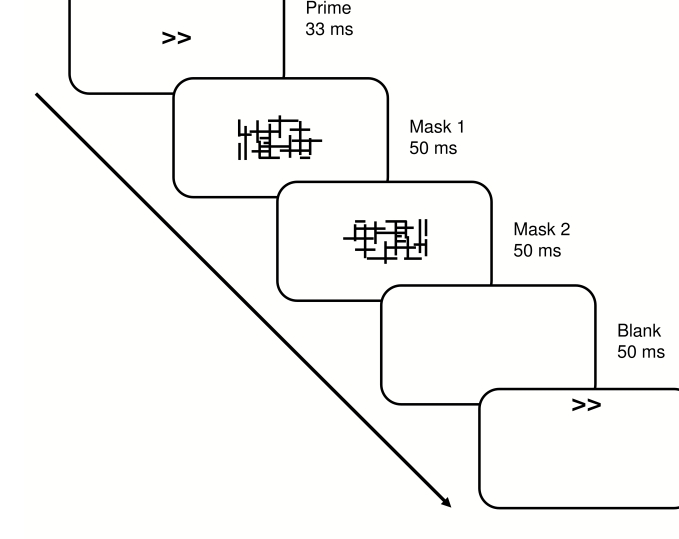
Stimulus- and trial-structure in Experiment 2.

The MP part of the experiment comprised 5 blocks of 60 trials each, and the FC part comprised 2 blocks of 80 trials. Each part began with a 20-trial practice block. Trial structure in the MP part is depicted in Figure 4. Each trial consisted of a 33-ms prime, immediately followed by a 50-ms mask, which in turn was followed immediately by a second 50-ms mask (‘flicker mask’). Fifty ms after offset of the second mask, a target was presented for 100 ms. Primes and masks were presented at fixation, targets were presented at fixation, 2° above fixation, or 2° below fixation.

ITI was 1,300 ms. On compatible trials, prime and target arrows pointed in the same direction. On incompatible trials, they pointed in opposite directions. All conditions (3 target positions x 2 compatibility levels) were equiprobable and randomized within each block. Participants were instructed to respond as quickly and accurately as possible with a left or right key press to left-pointing and right-pointing target arrows, respectively.

In the FC part, each trial consisted of a left- or right-pointing prime arrow, presented randomly and with equal probability for 17, 33, 50, or 67 ms, immediately followed by a flicker mask. No subsequent targets were presented, and ITI was 1,300 ms. Participants were instructed to press the key corresponding to the prime arrow’s direction, and to make a guess if they could not identify the prime clearly.

#### Data analysis

Repeated measures analyses of variance (ANOVAs) were computed on the percentage of correct responses in the FC identification task for the factor Duration (17, 33, 50, 67 ms). ANOVAs were computed on correct RTs and error rates for the factors Target Position (On Fixation, Off Fixation – collapsed across above- and below-fixation presentations) and Compatibility (compatible, incompatible) in the MP task.

### Results

**Figure 5.**
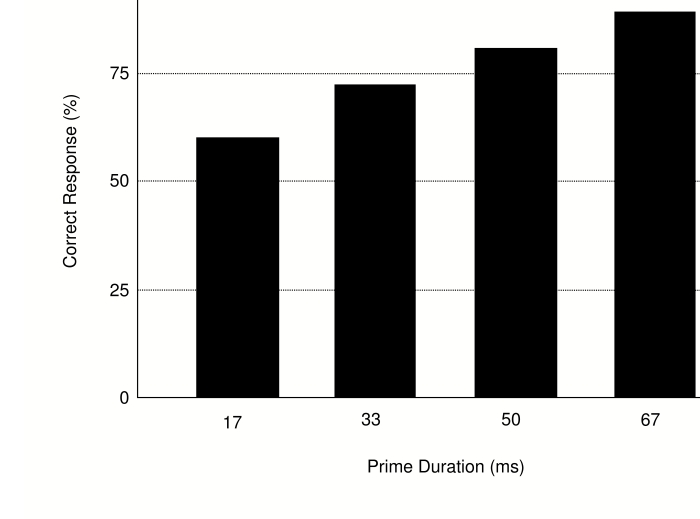
Forced Choice identification performance in Experiment 2.

Prime identification performance (Figure 5) increased with increasing prime duration, *F*(3, 39) = 28.65, *MSE* = 75.25, *p* < .001, but was significantly above chance (50%) for all prime durations, all *ts* > 3.3, all *ps* < .005.

Reaction times in the MP task were shorter for On-Fixation targets than for Off-Fixation targets, *F*(1, 13) = 4.90, *MSE* = 78.21, *p* = .045, and shorter on incompatible trials than on compatible trials (NCE), *F*(1, 13) = 6.36, *MSE* = 186.90, *p* = .024.

**Figure 6.**
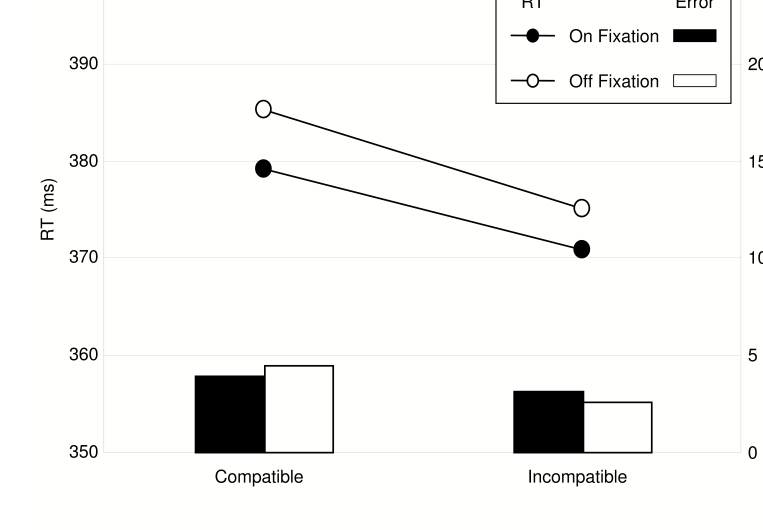
Reaction times (lines) and error rates (bars) in Experiment 2, plotted separately for On-Fixation (black) and Off-Fixation (white) targets.

Importantly, these factors did not interact, that is, NCEs of about the same magnitude were obtained for On-Fixation and Off-Fixation targets (see Table 2 and Figure 6).

**Table 2.** Priming effects (NCEs) on Reaction Times (ms) and Error Rates (%) on central and non-central targets in Experiment 1.

	RT (ms)			Error Rates (%)		
		95% CI			95% CI	
	NCE	lower	upper	NCE	lower	upper
Central Targets	-8.2	-17.7	1.2	-0.7	-2.9	1.4
Non-Central Targets	-10.2	-17.9	-2.5	-1.9	-3.9	0.2

Although Figure 6 indicates that error rates showed the same basic pattern as RTs, no significant effects were obtained, all *Fs* < 2.2, all *ps* > .16.

### Discussion

The results of Experiment 2 confirm the predictions of the self-inhibition hypothesis of masked priming: NCEs with irrelevant masks were obtained not only when primes, masks, and targets appeared at the same location, but also when targets appeared at a different location, and these effects were of approximately the same size regardless of target position (numerically, NCEs with non-central targets were larger than NCEs with central targets). The FC results and the priming effects with central targets almost exactly replicate the results reported by Schlaghecken and Eimer (2006). Importantly, however, the priming effects with non-central targets stand in direct contrast to Lleras and Enns’ (2006) results.

There are two main differences between the present experiment and the one reported by Lleras and Enns (2006). First, target position was randomized in the present experiment, but blocked (either all central or all non-central) in Lleras and Enns’ study. Thus in the present experiment, targets appeared at the same location as masked primes in one third of the trials. Therefore, it can be argued that primes and masks were spatially similar to targets *in principle*, that is, regardless of the actual target position on any given trial. If this argument is correct, then masks in the present experiment resembled targets in two (temporal *and* spatial) characteristics, and therefore were able to cause onset-triggered response suppression according to Lleras’ and Enns’ account.

The second major difference between the present experiment and those conducted by Lleras and Enns (2004, 2005, 2006) is the use of a flicker mask, whereas a continuous mask was employed in Lleras and Enns’ studies. As a consequence, prime identification performance was markedly reduced relative to the levels reported in Lleras and Enns (2004) (72% versus approximately 90% correct identification). According to these authors, it is unimportant whether or not the mask reduces prime visibility: NCEs can be obtained even with completely unmasked primes, provided an intervening stimulus (in this case, a ‘flanker’ rather than a ‘mask,’ see Lleras & Enns, 2006) contains response-relevant features. They argue that the correlation between mask density, prime visibility, and direction of priming effects – as reported, for example, by Eimer and Schlaghecken (2002) – is due to the fact that with masks composed of diagonal lines, a denser mask is more likely to contain arrow-like line intersections than a mask with less densely spaced lines. Therefore, the denser mask is more likely to facilitate reversed priming via *object updating*, not via self-inhibition triggered by the loss of sensory input to an existing motor activation, as argued by Schlaghecken and Eimer (2002, 2006). In the present experiment, however, the mask corresponded to Lleras and Enns’ (2004, 2005, 2006) *irrelevant mask* – that is, it only contained horizontal and vertical, but no diagonal lines, and thus should have been unable to facilitate object updating-induced NCEs, suggesting that there is another factor – possibly mask effectiveness – contributing to these effects.

It has to be noted, though, that in the present experiment – as in Experiment 1 – prime identification performance indicated that NCEs were obtained with primes that were clearly above the threshold of conscious perception (68 – 73% correct identifications on average). This contrasts with earlier findings showing that with identification levels of more than approximately 66%, NCEs begin to turn into PCEs (Eimer & Schlaghecken, 2002), suggesting a close link between the absence of conscious prime perception and the presence of NCEs (see also Klapp & Hinkley, 2002). However, those experiments differed from the present ones in one important aspect, as they used a staircase procedure to determine identification levels (i.e., after a correct response, prime identification on the next trial was made more difficult, after an incorrect response, it was made easier). The present experiments, in contrast, used a simple forced choice procedure. It might be argued that the former promotes a more careful response mode, whereas the latter promotes more spontaneous reactions, which therefore might be more susceptible to direct response priming effects.6 In this case, performance levels in the present experiments would systematically overestimate the actual level of prime identification and hence the primes’ perceptual strength.

On the basis of the present data, it is not possible to decide which of the two factors – target presentation or mask effectiveness – might be responsible for the difference between the present results and those reported by Lleras and Enns (2006). However, the self-inhibition account and the object-updating-plus-onset-triggered-suppression account make opposite predictions about the relative importance of these two factors: According to the former, mask effectiveness should affect priming effects, whereas target location should be largely irrelevant. According to the latter, the reverse should be true – priming effects should be independent of mask effectiveness, but NCEs should disappear when the mask is both geometrically and spatially dissimilar to the target. The following experiment was conducted to test these predictions.

## Experiment 3: More Irrelevant Masks and Non-Central Targets

In this experiment, no targets were presented at fixation, while other aspects of Experiment 2 were maintained. In particular, the same masking procedure and type of mask were employed as in the previous experiment. According to the self-inhibition account, NCEs should be obtained under these conditions. The updating-plus-onset-triggered-suppression account, in contrast, predicts PCEs.

A further difference between this experiment and the preceding one was an alteration in the FC procedure: In order to encourage a slower, less spontaneous response mode, the mask in the FC task was followed after 50 ms by two simultaneously presented question marks (presented above and below fixation). Participants were instructed not to respond before the question marks appeared. It was reasoned that this enforced delay would make responses less susceptible to direct motor priming. If identification performance in the previous experiments had in fact been artificially enhanced by motor priming effects, then identification levels in the present experiment should be substantially lower. In contrast, if similar identification levels were achieved, then this would support the notion that the NCEs in Experiments 1 and 2 had indeed been obtained with supraliminal primes.

### Method

#### Participants

Ten volunteers (four male), aged 19–41 years (M = 24.7), participated in the experiment. All but one participant were right-handed, and all had normal or corrected-to-normal vision.

#### Stimuli and apparatus

These were identical to Experiment 2.

#### Procedure

The procedure was similar to Experiment 2, with the following exceptions: a) no central targets were presented; b) block length was reduced to 40 trials each (without central targets, it was possible to reduce the number of trials by one third relative to Experiment 2); and c) in the FC task, the mask was followed after a 50-ms delay by two question marks (appearing at the same positions as the targets in the MP task), which remained on the screen until a response was given. Participants were instructed to respond only once the question marks had appeared.

#### Data analyses

For the FC data, the same analyses were carried out as in Experiment 2. RTs and error rates on compatible and incompatible trials in the masked prime task were compared using paired t-tests.

### Results

**Figure 7.**
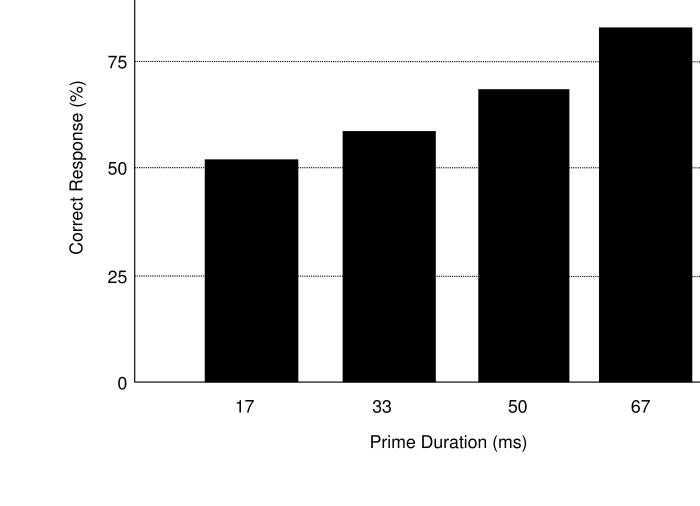
Forced Choice identification performance in Experiment 3.

**Figure 8.**
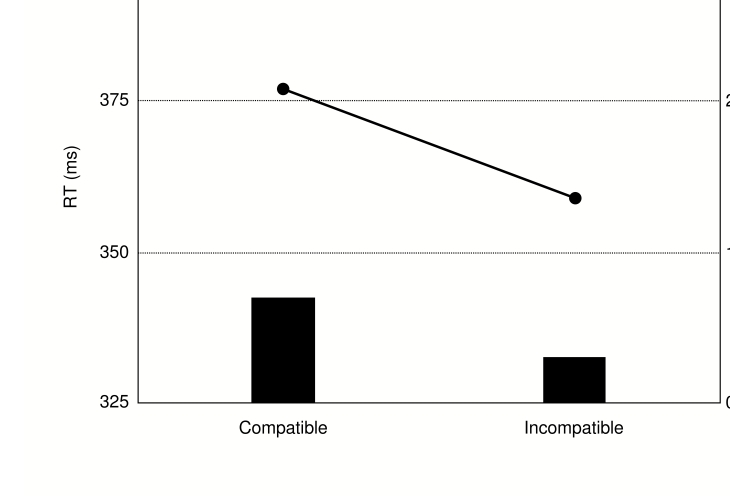
Reaction times (lines) and error rates (bars) in Experiment 3.

As expected, prime identification performancen (Figure 7) increased with increasing prime duration, *F*(3, 27) = 10.76, *MSE* = 162.96, *p* = .001. Importantly, however, identification performance was significantly above chance only for 50- and 67-ms primes, *t*(9) = 2.77, *p* = .022, and *t*(9) = 6.71, *p* < .001, respectively, but not for 17- and 33-ms primes, both *ts* < 1.7, both *ps* > .13. Reaction times were significantly shorter and error rates significantly lower on incompatible trials relative to compatible trials, *t*(9) = 3.01, *p* = .015 and *t*(9) = 2.47, *p* = .035, respectively (Figure 8).

#### Discussion

The present experiment aimed to recreate Lleras and Enns’ (2006) ‘flashed irrelevant mask, off-fixation target’ condition by masking arrow primes with irrelevant masks (containing no diagonal, arrow-like lines) and by always presenting targets at a location distinct from the location of primes and masks. The main procedural difference between this and Lleras and Enns’ (2006) experiment was the use of a flicker mask, which drastically reduced prime visibility.

According to the object-updating-plus-onset-triggered-suppression hypothesis, the present experiment should have yielded PCEs, replicating Lleras and Enns’ (2006) results. Clearly, this was not the case. Instead, significant NCEs were obtained, as predicted by the self-inhibition account. According to this hypothesis, NCEs should occur when the initial (low-level) motor activation triggered by the prime is suddenly no longer supported by sensory evidence, regardless of geometrical, spatial, or temporal similarities between primes, masks, and targets. The present experiment confirmed this prediction. Of course, this is not to deny that such similarities systematically influence priming effects – they obviously do, as suggested by common sense and as demonstrated in numerous studies (e.g., Jaśkowski & Przekoracka-Krawczyk, 2005; Lleras & Enns, 2004, 2005, 2006; Verleger et al., 2004). However, the present results strongly suggest that there is an additional inhibitory process in masked priming which is independent of these factors (i.e., low-level self-inhibition).

A further aim of this experiment was to investigate whether the relatively high identification performance in the previous two experiments might have been due to direct motor priming. By preventing participants from responding too quickly, identification performance for short-duration primes was indeed found to be dramatically reduced and no longer significantly different from chance. In contrast, identification performance for long-duration primes remained above chance level (though performance levels for 50-ms primes – but not for 67-ms primes – still appeared reduced in the present experiment relative to Experiment 2). This suggests that performance levels for the longest primes might reflect processes different from those that determine performance levels for shorter primes. These results are in line with the assumption that performance with short-duration primes is at least in part determined by direct motor priming, whereas performance with long-duration primes in Experiments 1 and 2 actually reflects conscious prime identification.

## General Discussion

A current debate in the masked priming literature concerns the question whether NCEs – shorter RTs and lower error rates on incompatible relative to compatible trials – reflect a self-inhibition mechanism in low-level motor control, or whether they are ultimately triggered by the mask, provided this stimulus is sufficiently similar to primes and targets with respect to geometrical, spatial, and/or temporal features. In previous studies, NCEs were obtained when masking stimuli met at least two of these three criteria, whereas only PCEs – shorter RTs and lower error rates on compatible relative to incompatible trials (i.e., ‘normal’ priming effects) – were obtained when this was not the case (e.g., Jaśkowski & Przekoracka-Krawczyk, 2005; Lleras & Enns, 2004, 2005, 2006; Verleger et al., 2004). These results have been interpreted as disproving the self-inhibition hypothesis and supporting (variants of) the hypothesis that the NCE is mask-induced. Furthermore, Verleger and colleagues (2005) have argued that arrows are ‘special’ and are more suited than other stimuli to trigger NCEs when employed as primes and targets.

In three masked priming experiments, the present study demonstrated that NCEs can be obtained with stimuli other than arrows (Exp. 1), and with irrelevant masks and targets that appear at a different position from the masked primes (Exp. 2 & 3). These results clearly contradict the predictions of the mask-induced NCE hypotheses, but are fully in line with the self-inhibition account of masked priming.

 It should be stressed that this conclusion does not deny the existence of mask-induced effects. Obviously, such effects not only exist, but can have a dramatic impact on overt performance as demonstrated, for example, by Jaśkowski and Przekoracka-Krawczyk (2005), who obtained NCEs with ‘masking’ stimuli that did not reduce prime visibility, but did contain arrow-like stimuli (see also Lleras & Enns, 2006). The mechanisms underlying such mask-induced reversal of primed motor activation are as yet not fully understood, and future studies should explore this phenomenon in more detail. In the context of the present experiments, however, the central argument is that the existence of mask-induced NCEs is independent of the existence of self-inhibition-induced NCEs: Both might be obtained in the same experimental paradigm, but it is also possible to obtain the former under conditions that disallow self-inhibition (e.g., with unmasked primes), and to obtain the latter under conditions that exclude the possibility of mask-induced NCEs (according to the criteria set out by the proponents of these accounts). 

Thus in our view, the mask-induced NCE account and the self-inhibition NCE account are not mutually exclusive, but simply describe different processes that result in the same observable effect (the same argument has also been made by Klapp, 2005). Interestingly, though, it seems that some proponents of mask-induced NCE-accounts disagree with this view, arguing that no low-level self-inhibition processes are involved at all (e.g., Jaśkowski & Przekoracka-Krawczyk, 2005; Lleras & Enns, 2006). This might point to an important theoretical difference between these two approaches regarding the question whether inhibitory control is possible even at low, ‘automatic’ processing levels, or whether it always requires higher-level executive commands. Given that opponent processing – the mechanism assumed to mediate self-inhibition (Bowman, Schlaghecken, & Eimer, 2006; Schlaghecken & Eimer, 2002) – appears to be a general processing principle of the central nervous system from perceptual input to motor output (e.g., Hurvich & Jameson, 1974; Levine & Leven, 1991; Pearson, 1993; Pribe, Grossberg, & Cohen, 1997), it seems reasonable to assume that this principle characterizes low-level perceptuo-motor control processes as well. On the other hand, however, it has frequently been argued that inhibitory control is a typical higher-level cognitive control function of central executive processes mediated by the prefrontal cortex (e.g., Band & van Boxtel, 1999; Faw, 2003). In line with this reasoning, evidence has been provided that inhibitory control is available only with supraliminally presented stimuli, but not with subliminally presented stimuli (e.g., Allport, Tipper, & Chmiel, 1985; Marcel, 1980; McCormick, 1997; Merikle, Joordens, & Stolz, 1995; Neill, Valdes, & Terry, 1995; Tsushima, Sasaki, & Watanabe, 2006). In fact, the NCE obtained with subliminal or near-threshold primes appears to be the only example where this general relationship does not hold, suggesting that this effect is either highly unusual, or simply not what it seems (i.e., not due to low-level inhibition).

However, we would like to argue that there is a third possibility. Recently, the argument has gained new momentum that the dichotomy between ‘low-level’ ‘automatic’ processes on the one hand and ‘high-level’ ‘controlled’ processes on the other is misleading (see, e.g., Hommel, 2007). In fact, common sense suggests that processes that appear to be ‘high-level’ do in fact have to be instantiated by ‘low-level’ mechanisms if the homunculus fallacy is to be avoided. In this context it is important to reiterate that the self-inhibition account does not make any claims about the role of participants’ conscious awareness of the prime. In fact, the perceptual learning study mentioned in the Discussion of Experiment 1 (Schlaghecken et al., in press) found that although training drastically improved both FC prime identification performance and subjective prime awareness, it had no effect on NCEs triggered by the masked primes. This strongly indicates that the physical characteristics of primes and masks, but not the presence or absence of conscious prime perception itself, determine the direction of priming effects.

If this is the case, however, it also means that performance in FC tasks can only be used to roughly estimate the influence of prime and mask on the motor system. Obviously, this comes dangerously close to the possibility of an ‘unfalsifiable’ argument (see also Lleras & Enns, 2006): When NCEs are obtained in a standard MP task, then it is assumed that the prime triggered a strong initial activation and that the mask was effective in rapidly removing the sensory evidence from the motor system. When no NCEs are obtained in this paradigm, then either the mask was ineffective, or the prime-induced activation was too weak to warrant self-inhibition. In other words, any outcome can be explained by making certain post-hoc assumptions about primes and masks. If FC prime identification or detection performance is not a suitable measure of the prime’s impact on the motor system – and we argue that it is not – then this problem can not be solved without an additional, independent measure of low-level motor activity. Future studies will have to address this issue in order to clarify the nature of the NCE and to investigate the possibility of low-level inhibitory perceptuo-motor control.
